# Strategic Framework for Additive Manufacturing with Smart Polymer Composites: A Pathway to Net-Zero Manufacturing

**DOI:** 10.3390/polym17101336

**Published:** 2025-05-14

**Authors:** Alok Yadav, Rajiv Kumar Garg, Anish Sachdeva, Karishma M. Qureshi, Mohamed Rafik Noor Mohamed Qureshi

**Affiliations:** 1 Department of Industrial and Production Engineering, Dr. B. R. Ambedkar National Institute of Technology, Jalandhar 144008, India; aloky.ip.21@nitj.ac.in (A.Y.); gargrk@nitj.ac.in (R.K.G.); asachdeva@nitj.ac.in (A.S.); 2 Department of Mechanical Engineering, Parul Institute of Technology, Parul University, Waghodia 391760, India; kariq18@gmail.com; 3 Department of Industrial Engineering, College of Engineering, King Khalid University, Abha 61421, Saudi Arabia

**Keywords:** polymer-based smart materials, additive manufacturing, net-zero manufacturing, sustainability, challenges

## Abstract

Despite manufacturing firms recognizing the potential benefits of polymer-based smart materials (PBSM) in additive manufacturing (AM), their large-scale integration remains limited. As manufacturing firms strive toward net-zero emissions (NZE) and sustainable manufacturing, integrating PBSM into AM could be pivotal for manufacturing firms striving to achieve NZE and more sustainable production. In this regard, this study uses a mixed-method approach: a systematic literature review (SLR) to address the current trends and critical challenges associated with the “development, processing, and scalability” of PBSM adoption for AM. Further, the study analyzes 100 responses from Indian manufacturing firms, employing exploratory factor analysis (EFA) to develop a framework. This framework is further validated by determining the priority order of challenges using the Combined Compromise Solution (CoCoSo) through a case study. The outcome highlights that end-of-life management and lack of standardization are the most critical challenges for manufacturing firms, restricting the adoption of PBSM for AM. This research provides valuable insights for industry professionals and academia, guiding a strategic roadmap toward net-zero manufacturing. With this transformation, industries can align with global net-zero targets and contribute to India’s net-zero economy (NZE) goal by 2070.

## 1. Introduction

The rapid advancement of science and technology has played a key role in developing polymer-based smart materials (PBSM), which integrates principles from materials science, nanotechnology, chemistry, and engineering [1]. These materials exhibit adaptive properties, such as self-healing, shape memory, and electroactivity, making them ideal for high-performance applications [2]. Additive manufacturing (AM) has emerged as a transformative fabrication technique that enhances the sustainability and functionality of smart materials by enabling precise, cost-effective, and waste-reducing production [3,4]. AM provides the precise, layer-by-layer fabrication of complex shapes while minimizing material waste. AM offers many environmental advantages compared to conventional manufacturing, including minimized raw material consumption, minimum energy usage, and reduced machine emissions [5,6]. PBSM is important in advancing sustainable manufacturing, particularly in achieving net-zero emissions in manufacturing [7,8]. Their integration into AM technologies facilitates energy-efficient production, minimizes material waste, and enables the design of lightweight, high-strength structures. AM has built the customized manufacturing of these materials with improved mechanical, electrical, and thermal properties, driving innovation across aerospace, biomedical, and industrial applications [9,10,11]. Smart materials like shape-memory polymers (SMPs) and shape-memory alloys (SMAs) have a remarkable ability to remember and return to their original shape when triggered by environmental changes [12]. This unique property makes them especially useful in sustainable manufacturing, where they can be used in active disassembly [13]. In this process, these materials act as intelligent fasteners that automatically detach when exposed to specific external conditions, making recycling and reusing components much more efficient [14].

The integration of smart materials further enhances their sustainability potential. As firms are moving towards striving to reduce their negative environmental impact, the demand for sustainable AM solutions is increasing [15,16]. Government and policymaker leaders are encouraging innovation to minimize waste, improve workplace sustainability, and explore novel materials for sustainable manufacturing [17]. These efforts are driving the development of value-added components and net-zero manufacturing practices.

Despite these potential benefits, many challenges are associated with adopting PBSM in AM [18,19]. These include the need for improved material formulations, enhanced printability, and the development of scalable, high-performance manufacturing techniques. Sustainability concerns, such as recyclability, biodegradability, and the environmental impact of polymer-based AM processes, must be addressed to align with net-zero goals [20,21].

Instead of increasing interest in PBSM for AM, there is a lack of comprehensive studies identifying emerging trends and advancements in this field. Critical challenges, such as maintaining material properties during AM processing, standardization issues, and scalability constraints, remain underexplored, restricting the adoption of polymer-based smart materials for AM for sustainable manufacturing practices [8,22]. Prioritizing these challenges in the context of net-zero manufacturing is limited, making it difficult to establish effective mitigation strategies. Therefore, this research aims to address the following research questions (RQ):

RQ 1. To review and analyze the emerging trends in the literature on PBSM and AM in the context of net-zero manufacturing.

RQ 2. To identify, validate and prioritize the critical challenges of PBSM practices for AM to support net-zero manufacturing goals.

RQ 3. To develop a framework for manufacturing firms to achieve net-zero manufacturing.

This research starts with an introduction to smart materials and their role in net-zero manufacturing. It then explores key AM techniques for processing PBSM, emerging trends, and future research directions. After that, the study examines challenges related to adopting PBSM in AM. We employ exploratory factor analysis (EFA) to develop a framework. This framework is further validated by determining the priority order of challenges using the Combined Compromise Solution (CoCoSo) through a case study and supporting their responsible adoption in AM for achieving net-zero goals.

This research is structured into six Sections. Section 2 highlights the literature review. Section 3 explores the research methodology. Section 4 outlines the results of the study. Section 5 discusses the outcomes of the results. In Section 6, we provide the implications of the study. Finally, Section 7 concludes the study with limitations and future scope.

## 2. Literature Review

PBSM are advanced polymers designed to be environmentally friendly throughout their lifecycle, often derived from renewable resources or engineered for biodegradability. These materials possess unique properties that allow them to respond to external stimuli (like temperature, pH, or light), making them highly functional in various applications [23]. In the context of AM, these smart materials improve the precision and efficiency of 3D printing by enabling the creation of complex structures and reducing material waste [24]. For example, SMPs can be printed into adaptive structures that change shape in response to environmental conditions, making them valuable for applications in biomedical devices, aerospace, and energy-efficient systems. The integration of PBSM into AM plays a key role in advancing sustainability and achieving NZE within manufacturing. AM reduces material waste by depositing material only where needed, and when we use biodegradable or recyclable polymers in AM, it also reduces environmental emissions.

The energy efficiency of smart materials (like self-healing polymers) increases the product’s lifetime and minimizes resource consumption. Also, replacing petroleum-based polymers with sustainable alternatives reduces emissions associated with material manufacturing and disposal, supporting the global shift toward net-zero manufacturing [25,26]. By using these materials in AM, firms can develop innovative, high-performance solutions while reducing their environmental emissions and aligning with sustainability goals.

**Figure 1:**
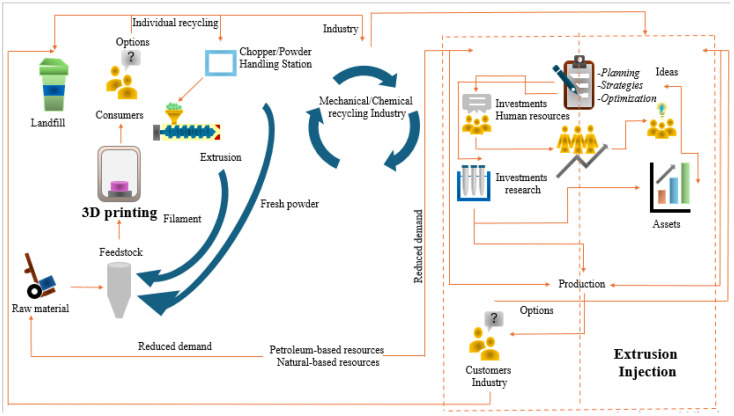
Integration of advanced polymer processing, recycling, and circular economy for net-zero manufacturing (adapted from [23]).

PBSM used in AM provides significant potential for recycling and reuse, contributing to sustainable manufacturing. High-value secondary raw materials from recycled polymers can minimize environmental emissions, aligning with net-zero goals [27]. AM faces challenges in integrating recycled polymers due to different polymer properties, processing barriers, and mechanical performance concerns [28]. In this regard, compatibility between advanced AM technologies and existing recycling infrastructures is important. A key challenge remains: “Can AM processes be optimized to use PBSM without compromising performance and functionality?”. Therefore, Figure 1 represents a comprehensive approach linking modern polymer processing, recycling strategies, and circular economy integration to drive net-zero manufacturing.

### 2.1. The Growth of Polymer 3D Printing

**Figure 2:**
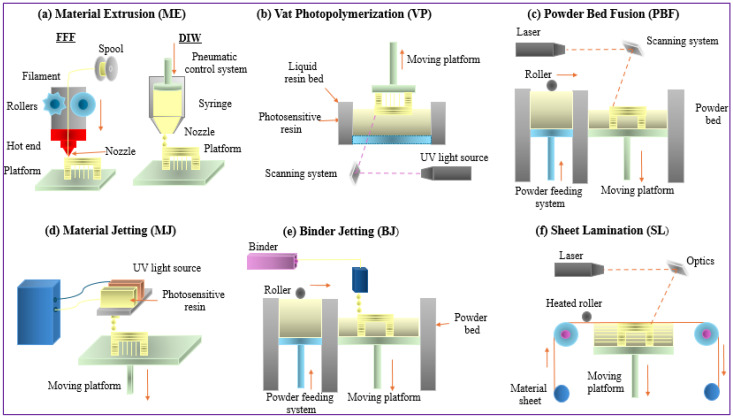
Evolution of polymer-based AM in six panels, grouped into three stages: (**a**,**b**) Rapid prototyping; (**c**,**d**) Pilot/low-volume production; (**e**,**f**) Emerging micro-factories for scalable, customized manufacturing (adapted from [1]).

The evolution of polymer 3D printing is a story of innovation, shaping AM through material advancements. While the concept of AM dates back over 50 years, its practical emergence began in the 1980s with the commercialization of stereolithography in 1987. The following decades saw breakthroughs like fused filament fabrication (FFF) and selective laser sintering (SLS), fueling rapid industrial adoption. As early patents expired in the 2000s, 3D printing became widely accessible, revolutionizing industries like aerospace and medicine [29]. Stereolithography, the first 3D printing technique, pioneered vat polymerization by solidifying resin to form polymer components. Polymers are integral to 3D printing, progressing from fragile early versions to advanced, high-performance materials. Common thermoplastics like PLA and ABS dominate filament-based systems, while polyetheretherketone (PEEK) and Polyetherketoneketone (PEKK) are gaining popularity for their superior properties [30]. Nylons and TPU are extensively used in powder bed fusion, and thermosetting polymers, once limited to vat polymerization, are now being adapted for extrusion and selective laser sintering. These polymer materials are available in diverse forms, including filaments, pellets, liquid resins, and powders, enabling a wide range of AM applications [31]. Initially, AM adapted existing polymers, but recent advancements focus on developing high-performance materials tailored for 3D printing. Intelligent polymers, shape memory polymers, and smart hydrogels enable responsive and adaptive functionalities. In contrast, liquid crystal polymers (LCPs) and liquid crystal elastomers (LCEs) have paved the way for 4D printing, where printed objects dynamically transform over time [1,32]. This integration of smart materials with 3D printing has unlocked new possibilities in robotics, biomedical devices, and next-generation manufacturing, pushing the boundaries of innovation. Figure 2 shows how polymer-based AM has progressed beyond its origins in rapid prototyping toward more robust production workflows. In Stage 1 (a–b), firms leverage AM for quick design iterations, producing single units of concept models or fit-checks with common thermoplastics, such as PLA and ABS. Transitioning to Stage 2 (b–c), small pilot runs using engineering-grade polymers (e.g., PEEK, PEKK) test performance in real-world environments. Stage 3 (e–f) remains aspirational for many SMEs: here, flexible “micro-factory” setups combine automated AM cells with post-processing and inline quality control to manufacture hundreds or thousands of customized parts per month.

### 2.2. Design for AM and Net-Zero Potential

**Figure 3:**
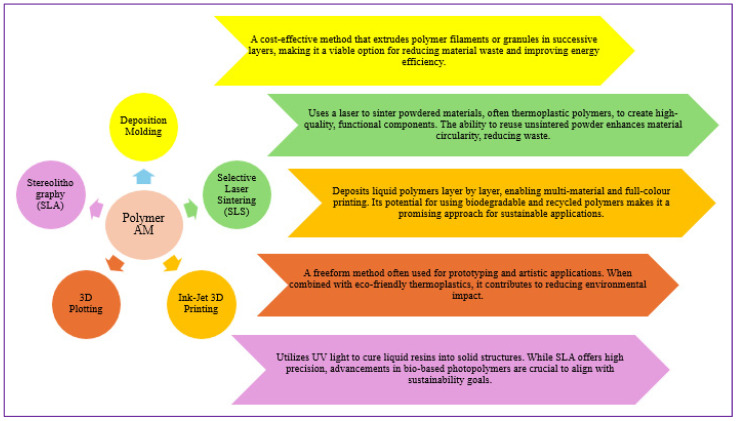
Strategy to enhance net-zero manufacturing using 3D printing technique (Author’s work).

Three-dimensional printing technology, driven by digital models, enables the creation of physical objects through the precise layering of materials, offering a sustainable alternative to conventional manufacturing. Many polymer-based 3D printing methods, including deposition molding, selective laser sintering (SLS), ink-jet 3D printing, stereolithography (SLA), and 3D drawing, each present unique advantages and challenges in pursuing net-zero emissions. The selection of an appropriate 3D printing technique is critical, considering factors, such as material efficiency, energy consumption, waste reduction, and recyclability, which are all essential for sustainable manufacturing [33]. The transition to NZE in polymer 3D printing requires continuous innovation in material development, energy-efficient printing processes, and recycling strategies. As advanced technologies drive improvements in energy consumption, recyclability, and bio-based polymer use, 3D printing holds immense potential to revolutionize sustainable manufacturing [34]. Figure 3 represents the strategy to enhance net-zero manufacturing using 3D printing and its contributions to net-zero manufacturing goals.

### 2.3. Polymer Fabrication Techniques

Polymer fabrication techniques shape polymeric materials into the desired form with the help of various methods like molding (stamping, injection molding, thermoforming), vacuum forming, and extrusion. These techniques are ideal for mass production due to their cost efficiency, high speed, and consistent quality. Unlike AM, formative methods excel in the large-scale production of complex, uniform, and aesthetically refined polymeric parts. Like 3D printing, injection molding and extrusion generate polymeric waste, making integrating recycled materials essential for net-zero manufacturing. Advanced extrusion systems incorporate regrind technology, ensuring seamless recycling of secondary raw materials. A hybrid approach combining 3D printing’s precision with the scalability of injection molding and extrusion offers new possibilities for polymeric product development [7,35]. Achieving net-zero manufacturing requires digitalization, automation, and workforce training advancements. These innovations enhance precision, minimize waste, optimize additives, improve energy efficiency, and enable toxin-free material production. Comparing injection molding with other techniques highlights sustainability challenges. Direct inkjet 3D printing depends on solvents, increasing complexity, energy use, and environmental impact. Stereolithography uses toxic resins, requiring solvent-based post-processing. Due to greater material density, injection molding generally yields higher tensile strength and elongation at break than fused deposition modeling.

### 2.4. AM and Polymer-Based Smart Materials

AM of PBSM has revolutionized industrial and domestic applications, enabling rapid prototyping and complex geometries. The increasing demand for AM has driven the development of advanced polymer materials, including thermoplastics, thermoplastic elastomers, polymer powders, and bio-based resins. This rapid expansion has also raised concerns about sustainability, waste generation, and environmental impact. Table 1 outlines the summary of existing studies in AM and PBSM.

#### Characteristics and Properties

Smart polymers in AM exhibit unique stimuli-responsive behavior, biocompatibility, and mechanical strength, making them ideal for advanced applications. These materials adapt to external triggers, such as temperature, pH, light, and magnetic fields, enabling functionalities like self-healing, controlled drug delivery, and shape-memory effects. Their biocompatibility ensures safe integration into biomedical devices, with surface modifications enhancing cell adhesion, tissue regeneration, and biodegradability. Their mechanical strength, high tensile properties, elasticity, and fatigue resistance make them suitable for prosthetics, implants, and soft robotics. These properties position smart polymers as key materials for sustainable and high-performance 3D-printed components, driving innovation and net-zero potential in manufacturing. Table 2 provides a detailed summary of the characteristics and properties of smart polymers.

### 2.5. Literature Gaps

Despite the increasing interest in PBSM for AM, significant gaps remain in the literature. Existing studies primarily focus on material synthesis, properties, and laboratory-scale applications, with limited exploration of real-world adoption challenges in manufacturing environments. While PBSM exhibits self-healing, shape-memory, and stimuli-responsive properties, its printability, scalability, and recyclability challenges hinder large-scale implementation. The prioritization of these challenges in the context of net-zero manufacturing remains unexplored. The existing studies rarely integrate industry perspectives or real-world case studies. There is a lack of empirical studies that evaluate how the manufacturing sector can overcome economic and technical challenges to adopt PBSM for sustainable AM. End-of-life management and recycling of PBSM are also underexplored, which limits the potential for net-zero manufacturing. To address these gaps, this research provides a comprehensive analysis of PBSM adoption in AM, prioritizes the critical challenges, and proposes a strategic framework for overcoming these challenges.

### 2.6. Novelty of the Studies

This research uniquely bridges the gap between PBSM development and its real-world adoption in AM for net-zero manufacturing. As existing studies mainly focus on material properties and experimental validation, this study provides a systematic ranking of adoption challenges through a structured framework. The potential contributions include the following:

1. Categorization of critical challenges using EFA.
2. Prioritizing critical challenges based on industry impact using the CoCoSo method.
3. Practical insights for net-zero strategies, linking PBSM adoption with sustainability-driven manufacturing.

By integrating a systematic literature review, expert insights, and empirical validation, this study delivers a comprehensive roadmap for overcoming technical, economic, and regulatory challenges, enabling wider industrial adoption of PBSM in AM.

## 3. Research Methodology

This study uses a mixed-method approach to achieve the research questions, integrating systematic literature review (SLR) analysis, expert consultations, and input from six experts during the questionnaire design. A large-scale survey was conducted in 100 Indian manufacturing industries to validate the identified critical challenges through SLR that restrict the adoption of PBSM to support AM in the context of sustainability and net-zero manufacturing. Furthermore, through EFA, we grouped the identified challenges into clusters. The CoCoSo technique is used to assign weights and determine the priority of these challenges. This integrated approach collectively provided valuable insights into the prioritization of these challenges.

### 3.1. Systematic Literature Review Protocols

**Figure 4:**
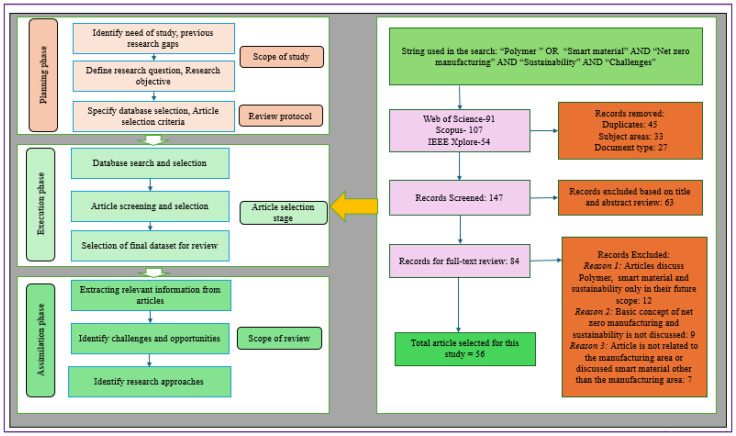
An SLR protocol for this study (Author’s work).

We conducted an SLR to identify the critical challenges of PBSM adoption for AM in the context of net-zero manufacturing, following the detailed protocols presented in Figure 4. This review identified 14 critical challenges, which are presented in Table 3.

### 3.2. Survey Details and Data Collection

In the initial phase of this study, an empirical investigation was conducted to statistically validate and provide a theoretical basis for the challenges identified through the SLR. A structured questionnaire was developed using a five-point Likert scale (ranging from 1-strongly disagree to 5-strongly agree), drawing upon prior studies in the field, particularly the work of [50]. To ensure clarity and relevance, the questionnaire underwent pre-testing by three academic experts from the Industrial and Production Engineering Department, as well as three industry professionals affiliated with leading manufacturing firms engaged in AM (refer to Table 4). Following in-depth discussions with these experts, several refinements were made in response to their suggestions. The sampling method and criteria used to select the participating organizations are described in detail below.

*Sampling Process and Eligibility Criteria:* A systematic sampling strategy was adopted to identify and select relevant manufacturing organizations for inclusion in this study. The process was carried out through the following structured steps:

- Sampling Frame: Initially, a comprehensive list of manufacturing organizations was compiled using industry databases and government records. This list formed the foundation for selecting potential participants for the study.

- Inclusion Criteria: Organizations were included in the study if they met specific criteria, such as having a functional website providing up-to-date information about the company, and if they were small- to medium-sized manufacturers that had either adopted or were in the process of adopting AM, particularly those related to the net-zero economy goal. These criteria ensured that the selected organizations were relevant to the research questions.
- Exclusion Criteria: Organizations were excluded from the study if they did not meet the inclusion criteria, if they were unwilling to participate, or if their data were incomplete or unreliable. This step was necessary to maintain the quality and integrity of the data.

A list of 511 manufacturing firms located across different regions of India was initially compiled from industry directories and contacted via email and professional networks over a three-month period (January–March 2025). In the first month, we received 63 responses. To boost participation, reminder emails and LinkedIn outreach yielded an additional 47 responses. After data cleaning (removing 10 incomplete submissions), 100 valid responses remained for analysis (corresponding to a 19.6% response rate). This response rate is typical for industry surveys and provides a sufficient sample size for factor analysis [51]. The actual sample size of 100 exceeded the minimum required sample size of 70 or higher (respondent-to-factor ratio of 5:1) for the proposed conceptual framework [52,53]. SME respondent demographics are presented in Table 5.

We conducted expert consultations and validated the classification using EFA to categorize the challenges. The 14 challenges were grouped into four categories: (1) Technological Challenges, (2) Environmental and Sustainability Challenges, (3) Economic and Market Challenges, and (4) Regulatory and Standardization Challenges. The survey results confirmed the significance of all challenges in the Indian manufacturing context, with mean scores, factor loading, and communalities for each challenge in Table 6. Furthermore, analysis showed that all study variables had factor loadings above 0.5, ensuring convergent validity. The Kaiser–Meyer–Olkin (KMO) value was 0.862, confirming the data’s suitability for factor analysis [52]. Further, to rank the 14 challenges, we applied the CoCoSo technique, which is discussed in the next Section.

### 3.3. Combined Compromise Solution (CoCoSo)

CoCoSo was proposed by Yazdani and is one of the recent MCDM techniques. This method is based on the combination of simple additive weighting (SAW) and an exponentially weighted product model. This method is effective in dealing with the ranking of the challenges [54,55]. The steps of the CoCoSo are provided in detail as follows:

Step 1: Initially, the linguistic assessment matrix is developed using linguistic terms for the evaluation criteria, using a five-point Likert scale (i.e., 1-Extremely Disagree; 5-Extremely Agree). Further, this matrix is converted into an initial decision-making matrix. The matrix [Y] *m* × *n* shows the initial decision-making matrix, including the m-number of challenges and n-experts. The matrix ‘*xij*’ element represents the performance of the challenges concerning the *j*th expert. In this study, the alternative is the ‘practices, and the criterion is the experts. In this context, ‘*xij*’ shows the importance of the *i*th practice as per the *j*th expert.

(1) $$Y_{ij}=\left[\begin{array}{ccc} y_{11} & \ldots & y_{1n}\\ \vdots & \ddots & \vdots \\ y_{m1} & \ldots & y_{mn} \end{array}\right];\;i=1,2,\ldots n,\;j=1,2,\ldots \ldots m$$

Step 2: The initial decision-making matrix is normalized using Equations (2) and (3).

For challenges, we will consider beneficial criteria:

(2) $$r_{ij}=\frac{y_{ij}-\min y_{ij}}{\max y_{ij}-\min y_{ij}};$$

For non-benefit/cost criteria:

(3) $$r_{ij}=\frac{{\max y_{ij}-y}_{ij}}{\max y_{ij}-\min y_{ij}};$$

Step 3: The weighted comparability sequence (*Si*) of each alternative and the power weight of comparability sequences (*Pi*) of each alternative are calculated using Equations (4) and (5), respectively.

(4) $$S_{i}=\sum_{j=1}^{n}\left(w_{j}r_{ij}\right)$$

(5) $$P_{i}=\prod_{j=1}^{n}{{(r}_{ij})}^{w_{j}}$$

Step 4: Relative weights of each alternative are calculated using three aggregation approaches, and the same is provided through Equations (6)–(8):

(6) $$k_{ia}=\frac{{S_{i}+P}_{i}}{\sum_{i=1}^{m}\left({S_{i}+P}_{i}\right)}$$

The Equation (6) shows the arithmetic mean of sums of scores, weighted sum measure (*Si*), and weight power measure (*Pi*)

(7) $$k_{ib}=\frac{S_{i}}{\min S_{i}}+\frac{P_{i}}{\min P_{i}}$$

Equation (7) delivers a sum of relative scores of the weighted comparability sequence (Si) and power weighted comparability sequence (*Pi*) compared to the best.

(8) $$k_{ic}=\frac{\lambda ({S_{i})+(1-\lambda )(P}_{i})}{\lambda \max S_{i}+\left(1-\lambda \right)\max P_{i}}$$

Equation (8) signifies the balanced compromise of weighted comparability sequence (*Si*) and power weighted comparability sequence (*Pi*) score. The value of the parameter λ is usually 0.5, or experts might choose it as per the requirements.

Step 5: The alternatives’ weight relies on the *ki* value, which is computed using Equation (9).

(9) $$k_{i}={\left(k_{ia}k_{ib}k_{ic}\right)}^{\frac{1}{3}}+\frac{1}{3}(k_{ia}+k_{ib}+k_{ic})$$

The final ranking of the challenges is provided in descending order of *ki* values, i.e., the challenges having the greatest value of *ki* are more critical.

## 4. Results and Discussion

This study employs a mixed-method approach that integrates (i) a systematic literature review to identify challenge themes, (ii) a practitioner survey to quantify their prevalence, and (iii) expert validation to ensure real-world applicability.

### 4.1. Main Information for Emerging Trends

The research in this field spans from 2013 to 2025, reflecting over a decade of scholarly contributions. Fifty-six documents have been published across 46 sources, including journals, books, and conference proceedings. The annual publication growth rate of 16.1% highlights the increasing interest in this area. The documents have an average citation count of 7.911 per paper, supported by 3235 references, indicating strong academic engagement. The average age of the documents is 2 years, suggesting that the field is relatively new and evolving rapidly.

Two hundred forty authors have contributed to this body of work, with an average of 4.48 co-authors per document, showcasing a high level of collaboration. However, only one single-authored document exists, emphasizing a preference for multi-author research efforts. The international co-authorship rate is 25%, reflecting significant global collaboration in advancing knowledge within this research area.

The distribution of document types includes 26 journal articles, five book chapters, 12 conference papers, 12 review articles, and one editorial piece, demonstrating a mix of theoretical research, practical applications, and scholarly reviews. This combination of publication types indicates that the field is well-supported by empirical research and comprehensive literature syntheses, making it a dynamic and growing study area.

#### Annual Publication Trends in This Research Area

**Figure 5:**
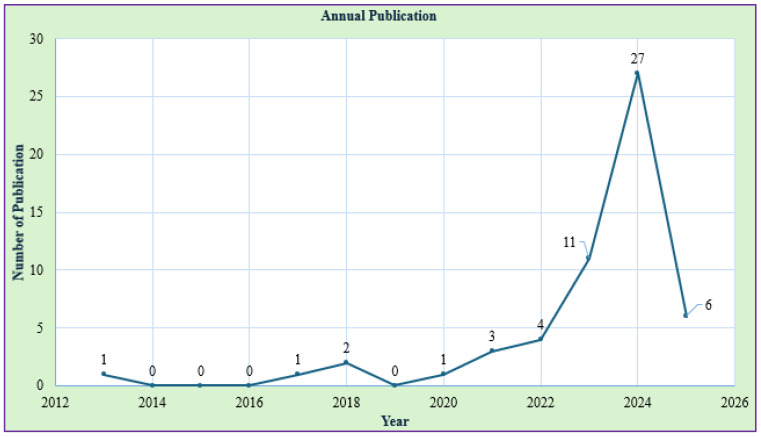
Yearly publications trends.

The publication trends (refer to Figure 5) reveal a gradual rise in research activity within this field. The first paper was published in 2013, followed by a gap between 2014 and 2016. Research interest grew slowly, with one paper in 2017, two in 2018, and one in 2020. A steady increase was observed in 2021 (three papers) and 2022 (four papers), marking the beginning of more consistent research efforts. A significant surge occurred in 2023, with 11 publications, followed by a peak in 2024, reaching 27 papers, indicating heightened research focus. In 2025, six papers have already been published, suggesting ongoing interest, although with some fluctuations. This trend highlights the rapid expansion of this research area, particularly in recent years.

### 4.2. EFA Outcomes

Through EFA, the identified challenges were categorized into four categories, with a total explained variance of 91.164%, leading to the development of a proposed framework for the manufacturing sector. Factor loadings ranged from 0.835 to 0.939, all exceeding the recommended threshold of 0.60. Also, commonalities ranged from 0.875 to 0.938, surpassing the acceptable limit of 0.50.

**Figure 6:**
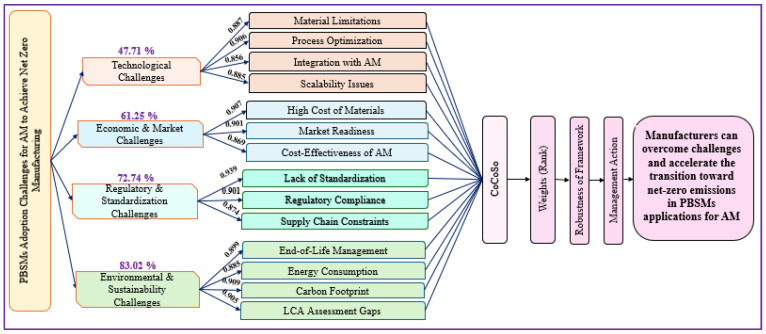
Farmwork for AM to overcome PBSM challenges to achieve net-zero manufacturing (Author’s work).

To clarify our factor-analytic approach, we conducted EFA using Principal Axis Factoring with Kaiser’s criterion (eigenvalues > 1) and applied an orthogonal Varimax rotation to maximize the interpretability of the four latent clusters. All 14 survey items exhibited communalities above 0.50 and factor loadings above 0.60, so no items were dropped. Based on EFA results, all the challenges are categorized into major clusters, and a framework for net-zero manufacturing is proposed and represented in Figure 6.

Each challenge group can be tackled in practice. Table 7 outlines each of the four clusters with example interventions and the primary stakeholders.

### 4.3. CoCoSo Outcomes

Table 8 shows initial decision-making matrix. Subsequently, Table 9 of normalized matrix, Table 10 of weighted normalized matrix and Table 11 of power of weighted compatibility matrix were calculated. Table 12 shows the final ranking of the challenges.

### 4.4. Sensitivity Analysis

**Figure 7:**
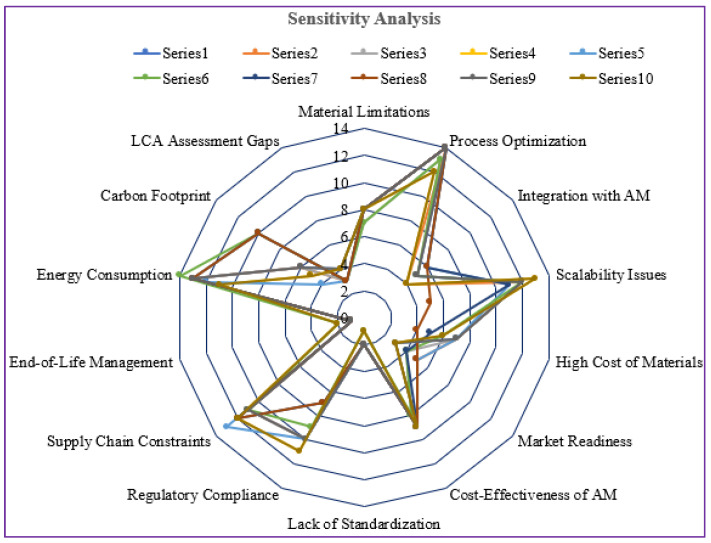
Results from the sensitivity analysis (Author’s work).

We conducted a sensitivity analysis to evaluate the robustness of the proposed framework. This analysis gives decision-makers insights into how variations in ratings and weights influence the prioritization of challenges. Since experts’ industry experience may influence their responses, potential biases were considered. Different weights were assigned to the identified challenges to ensure the framework’s robustness. In the analysis, we adjusted the ratings and weights of challenges identified through the CoCoSo method to observe any changes in their rankings. The sensitivity analysis explored scenarios where certain challenges gained, or lost influence based on input variations. The results indicate that even slight variations in the assigned weights significantly impact the final prioritization of challenges (refer to Figure 7). However, minimal changes were observed in the final ranking when different weights were applied, confirming the robustness of the proposed framework.

## 5. Discussion

While existing studies have detailed PBSM synthesis, properties, or single-industry case studies, none have systematically combined practitioner insights with multi-criteria ranking to prioritize adoption challenges. This study uniquely integrates: (1) a large-scale survey of Indian SMEs; (2) EFA-based clustering of critical challenges; and (3) CoCoSo ranking to generate a validated, practitioner-driven framework. This integrated SLR-EFA-CoCoSo approach bridges lab-scale materials research and real-world deployment, offering actionable guidance for manufacturers, policymakers, and researchers toward net-zero manufacturing.

### 5.1. Answer to RQ1- to Review and Analyze the Emerging Trends of the Literature in PBSM and AM in the Context of Net-Zero Manufacturing

This research conducted an SLR to explore the emerging research trends of PBSM in AM, mainly in the context of net-zero manufacturing. The analysis, covering publications from 2013 to 2025, revealed a significant annual publication growth rate of 16.1%, indicating an increasing focus on sustainable AM solutions. The area has expanded through author collaborations, with an average of 4.48 co-authors per document and 25% of research being internationally co-authored, showing global engagement in PBSM advancements. Key emerging trends include the shift from traditional polymer composites to functional, smart materials, such as shape-memory polymers (SMPs), self-healing polymers, and bio-based composites, which are increasingly explored for energy-efficient, sustainable AM applications. Despite these advancements, the literature reveals critical gaps in scalability, regulatory standardization, recyclability, and economic feasibility, restricting widespread industrial adoption.

### 5.2. Answer to RQ2- to Identify, Validate and Prioritize the Critical Challenges of PBSM Practices for AM

This research used an SLR, expert consultations, and empirical analysis to identify and rank the critical challenges associated with PBSM in AM for net-zero manufacturing. The final ranking of challenges (refer to Table 12) shows that End-of-Life Management, Lack of Standardization, and Market Readiness are the most significant challenges, underscoring sustainability, regulatory, and economic concerns. Technological limitations remain a major challenge, with material performance issues, process optimization complexities, and difficulties in scaling production impacting PBSM’s large-scale adoption. Many smart polymers struggle with thermal stability, mechanical strength, and AM compatibility, making industrial implementation challenging. Economic challenges, such as high raw material costs, uncertain cost-effectiveness, and lack of financial incentives, discourage widespread investment in PBSM-based AM solutions. The absence of global industry standards and evolving regulatory frameworks complicates integration into AM workflows. Supply chain constraints, high energy consumption, and uncertainties surrounding carbon footprint reduction are additional challenges that hinder the alignment of PBSM-based AM with net-zero goals. Addressing these issues requires targeted material innovations, stronger policy support, and financial incentives to accelerate the industrial adoption of PBSM for sustainable AM solutions.

### 5.3. Answer to RQ3- to Develop a Framework for Manufacturing Firms to Achieve Net-Zero Manufacturing

This study developed a comprehensive framework using EFA and the CoCoSo method to support manufacturing firms in achieving net-zero manufacturing. The framework systematically prioritizes key challenges and provides strategic solutions to overcome challenges related to technology, market adoption, regulatory constraints, and sustainability. A sensitivity analysis was conducted to check the robustness of the proposed framework (refer to Figure 7). This includes varying the weights and rankings of challenges identified through the CoCoSo method to determine the impact on prioritization. The results revealed that while minor variations in weights influenced rankings, the final prioritization remained stable, confirming the framework’s reliability in guiding manufacturing firms toward net-zero manufacturing goals.

The four challenge clusters identified by EFA, Technological, Environmental and Sustainability, Economic and Market, and Regulatory and Standardization, highlight the multifaceted barriers to PBSM adoption in AM. Technological Challenges, such as material limitations and integration issues, underscore the need for tailored polymer formulations and process parameters. Prior work demonstrates that optimizing AM settings for advanced polymers enhances printability and part performance, but scalability remains elusive without novel material process synergies [24,36]. Within Environmental and Sustainability Challenges, end-of-life management emerged as the main challenge. Efficient recycling routes for multi-phase smart composites are scarce, and legacy AM infrastructures often lack material-specific recovery pathways [46]. The Regulatory and Standardization Challenges, notably the absence of universally accepted standards for AM-printed sustainable materials, further constrain industrial uptake. Evolving environmental regulations demand adaptable compliance frameworks, yet standard test methods for smart polymer AM parts are still under development [48]. Harmonization of standards will be critical to ensure cross-industry interoperability and quality assurance. Economic and Market Challenges, such as market readiness and high material costs, reflect industry skepticism and uncertain return on investment. Empirical analyses show that SMEs often defer PBSM integration until raw-material prices decrease or financial incentives improve. Our strategic framework (Figure 6) directly addresses these issues by aligning innovation roadmaps with policy incentives, collaborative R&D, and scale-up strategies. Together, these insights validate our proposed roadmap for net-zero manufacturing, emphasizing targeted material innovation, robust LCA integration, standardization efforts, and economic de-risking to accelerate PBSM adoption in AM.

This result reflects a genuine industry shift rather than bias: manufacturers face mounting recycling regulations and customer demand for closed-loop products, and LCA research shows that without end-of-life solutions, upstream efficiency gains are lost to downstream waste. As a result, firms now treat robust recycling and biodegradability measures as prerequisites before optimizing AM processes.

The framework is adaptable beyond Indian SMEs, as the challenge categories are globally relevant. Non-Indian SMEs or large firms can easily apply it by adjusting the challenge priorities through local data and aligning actions with regional policies and stakeholders.

## 6. Implications of the Study

This research provides theoretical, practical, and policy implications for advancing the adoption of PBSM in AM to support net-zero manufacturing goals.

### 6.1. Theoretical Implications

This study expands the existing literature on PBSM in AM by systematically identifying and ranking critical adoption challenges using the EFA and CoCoSo methods. The study highlights gaps in scalability, standardization, and end-of-life management, providing a novel framework to bridge material science innovations with sustainable manufacturing practices.

### 6.2. Practical Implications

Manufacturers can leverage this study’s findings to optimize AM material selection and process efficiency. Firms can enhance economic feasibility and sustainability performance by addressing high material costs, energy consumption, and market readiness challenges. The study also underscores the need for collaborations between industries, research institutions, and policymakers to accelerate PBSM’s large-scale adoption.

### 6.3. Policy and Sustainability Implications

The research provides actionable insights for policymakers to establish standardization guidelines, financial incentives, and regulatory frameworks that support sustainable AM practices. By integrating recyclability, waste reduction, and carbon footprint assessment, policymakers can drive net-zero emissions adoption in manufacturing sectors. This study lays the foundation for advancing sustainable AM technologies and promoting wider industrial adoption of PBSM for net-zero manufacturing by addressing these multi-dimensional implications.

## 7. Conclusions, Limitations and Future Research Recommendations

In this study, we use a mixed-methods approach. Through a literature review, we identify the current trends, and the critical challenges related to PBSM integration into AM to achieve the net-zero goal within Indian Manufacturing SMEs. A survey of 100 Indian manufacturing firms was conducted to check the significance of these challenges, which were analyzed using EFA to identify four clusters and the CoCoSo method. The study ranked these challenges and developed a strategic framework to facilitate the transition to sustainable AM practices. Sensitivity analysis confirmed the framework’s robustness, ensuring its applicability in guiding manufacturers toward net-zero goals. The study’s outcomes highlight end-of-life management, lack of standardization, and market readiness as the most critical challenges to the large-scale adoption of PBSM in AM. This research demonstrates the importance of material innovation, regulatory support, energy-efficient AM technologies, and circular economy strategies in overcoming these challenges. By adopting these strategic approaches, manufacturers, policymakers, and researchers can accelerate the adoption of PBSM in AM, contributing to global sustainability targets and net-zero manufacturing goals.

The study primarily focuses on Indian manufacturing SMEs, limiting the generality of findings across different regions and industrial sectors. While the study integrates quantitative methodologies, the absence of real-world case studies restricts insights into practical implementation challenges. Future research should focus on empirical validation of the proposed framework by conducting real-world case studies in industrial settings to assess its practical feasibility. Integrating advanced technologies (like artificial intelligence, machine learning (ML), and digital twins) could optimize AM processes and improve the scalability of PBSM.

## Figures and Tables

**Table 1:** Summary of Key Studies on AM and PBSM.

Smart Materials	Methods	Results	Conclusions	Key Drawbacks	Reference
Shape-Memory Polymers (SMPs)	Fused Deposition Modeling (FDM) and Stereolithography (SLA)	Self-healing and shape-recovery with thermal/UV triggers	Enhances durability and sustainability in AM applications	Brittle output; requires post-cure; limited to thin sections	[31]
Polylactic Acid (PLA) Composites	3D Printing with bio-fillers	Improved mechanical strength and biodegradability	Polylactic acid (PLA)-based AM offer eco-friendly alternatives with strong performance	Reduced mechanical strength at high loads; moisture sensitivity	[36]
Polyurethane-Based Shape-Memory Polymer (SMPs)	Direct Ink Writing (DIW)	High elasticity and shape-memory response	Suitable for biomedical applications and flexible electronics	Low throughput; nozzle clogging; solvent handling issues	[31]
Carbon Nanotube-Reinforced Polymers	Selective Laser Sintering (SLS)	Enhanced electrical conductivity and mechanical properties	Ideal for advanced sensors and smart electronic components	Nonuniform dispersion; health/safety concerns; expensive feedstock	[37]
Bio-Based Resins	Vat Photopolymerization	High precision and recyclability	Supports sustainable AM with low environmental impact	Limited material choices; resin toxicity; high material cost	[38]
Thermoplastic Elastomers (TPEs)	Material Extrusion (ME)	High resilience and flexibility	Suitable for soft robotics and wearable devices	Poor dimensional accuracy; thermal degradation during extrusion	[15]
Graphene-Enhanced Polymers	Inkjet 3D Printing	Improved thermal stability and conductivity	Potential for energy storage and thermal management applications	Clogged printheads; limited print volume; high ink rheology requirements	[33]
Reversible Cross-Linking Polymers	Digital Light Processing (DLP)	Achieved self-repair and reusability	Enables circular economy approaches in AM	Slow build speed; high UV exposure; resin brittleness	[39]
Magneto-Responsive Polymers	Multi-Material 3D Printing	Controlled actuation under magnetic fields	Useful in biomedical implants and soft robotics	Complex calibration; reduced mechanical integrity at interfaces	[40]
Self-Healing Polymer Composites	Hybrid AM Techniques	Prolonged service life and reduced material waste	Enhances sustainability and functional durability in AM	Multi-step processing; longer cycle times; compatibility issues	[41]
4D-Printed Shape-Memory Polymers	Direct Ink Writing and FDM	Programmable shape transformation	Expands functionality in biomedical and aerospace applications	Limited repeatability; high material cost; intricate post-processing	[42]
Biodegradable Polymer Composites	Stereolithography and SLA	High strength and eco-friendly degradation	Supports sustainable AM for medical applications	Hydrolytic degradation in service; brittle when dry	[38]
Stimuli-Responsive Polymers	Multi-Material 3D Printing	Reacts to heat, light, and magnetic fields	Enables smart coatings and adaptive structures	Cross-contamination between materials; uneven layer adhesion	[43]
Conductive Polymers	Extrusion-based AM	Improved electrical conductivity	Ideal for wearable electronics and flexible sensors	Poor conductivity at scale; filler sedimentation; high viscosity	[21]

**Table 2:** Detailed Summary of Characteristics and Properties of Smart Polymers.

Characteristics	Property	Description	Key Materials	Mechanism	Applications
Stimuli-Responsive Behavior	Thermal Sensitivity	Polymers change phase or shape with temperature	Poly(N-isopropylacrylamide) (PNIPAAm), Polyurethane, PEG-based polymers	Lower Critical Solution Temperature (LCST) effect leads to volume change	Smart coatings, Drug delivery, Tissue engineering
	pH-Responsive Behavior	Structural change in response to acidity or alkalinity	Poly (acrylic acid), Chitosan, Poly (ethylene imine)	Protonation/deprotonation of functional groups	Oral drug delivery, pH sensors, Targeted cancer therapy
	Light-Responsive Behavior	Changes shape or fluorescence under light exposure	Spiropyrans, Azobenzenes, Photochromic hydrogels	Photoisomerization alters polymer conformation	Optical switches, Controlled drug release
	Magneto-Responsive Behavior	Responds to external magnetic fields	Iron oxide nanoparticles, Magnetic hydrogels	External magnetic fields induce movement or heat generation	Hyperthermia therapy, Remote-controlled drug release
Biocompatibility	Non-Toxicity	Polymers do not cause adverse biological reactions	PLA, PEEK, Hydrogels, Chitosan	Biodegradable and bioresorbable nature	Implants, Drug carriers, Scaffolds for tissue engineering
	Surface Modification for Biocompatibility	Enhances interaction with biological tissues	Titanium-coated polymers, Hydroxyapatite composites	Surface treatments promote cell attachment	Dental and orthopedic implants, Bone grafts
	Protein and Cell Adhesion	Supports cell growth and differentiation	Alginate, Collagen-based hydrogels	Chemical functionalization of polymer surfaces	Wound healing, Tissue scaffolding
	Biodegradability	Natural breakdown in biological environments	PLA, polyglycolic acid (PGA) and Polycaprolactone (PCL)	Enzymatic and hydrolytic degradation	Resorbable sutures, Drug-eluting implants
Mechanical Strength	Tensile Strength	Resistance to pulling forces	PEEK, Polyurethanes, PMMA	Polymer chain alignment affects tensile properties	Prosthetics, Load-bearing implants
	Elastic Modulus	Measures stiffness of material	PCL, Thermoplastic elastomers (TPU), Silicone elastomers	Higher cross-linking increases stiffness	Soft robotics, Flexible electronics
	Shape-Memory Effect	Ability to return to original shape after deformation	Shape-memory polymers (SMPs), Polyurethane blends	Thermo-responsive phase transition	Self-healing materials, Smart actuators
	Fatigue Resistance	Resistance to repeated mechanical stress	Thermoplastic elastomers, PEEK	Cross-linked polymer networks prevent microcracks	Wearable sensors, Aerospace components

**Table 3:** Challenges associated with PBSM adoption for AM.

Challenge	Description	Reference
Material Limitations	Limited availability of high-performance, sustainable polymer-based smart materials.	[44]
Process Optimization	Difficulty in optimizing AM parameters for sustainability and material efficiency.	[45]
Integration with AM	Challenges in effectively incorporating polymer-based smart materials into existing AM processes.	[24]
Scalability Issues	Difficulty in scaling AM processes for mass production using sustainable materials.	[8]
End-of-Life Management	Lack of efficient recycling and biodegradability strategies for smart polymers.	[46]
Energy Consumption	High energy demands in AM processes, impact sustainability goals.	[29]
Carbon Footprint	Uncertainty regarding the overall carbon footprint reduction in AM-based polymer materials.	[45]
LCA Assessment Gaps	Limited Life Cycle Assessment (LCA) studies for evaluating the environmental impact of polymer-based smart materials in AM.	[23]
High Cost of Materials	Sustainable smart materials often have higher production and procurement costs.	[13]
Market Readiness	Slow adoption of polymer-based smart materials due to industry skepticism and lack of awareness.	[47]
Cost-Effectiveness of AM	Unclear economic benefits of adopting AM for net-zero manufacturing.	[27]
Lack of Standardization	Absence of universally accepted standards for AM-printed sustainable materials.	[18]
Regulatory Compliance	Difficulty in meeting evolving environmental and industrial regulations.	[48]
Supply Chain Constraints	Limited availability and accessibility of sustainable raw materials for AM adoption.	[49]

**Table 4:** Details of the experts’ questionnaire pre-testing process.

No. of Experts	Role of Experts	Experience of Experts
Expert 1	Academia	32
Expert 2	Manufacturing head	18
Expert 3	Production Manager	9
Expert 4	Plant Manager	20
Expert 5	Production Engineer	22
Expert 6	R&D Manager	12

**Table 5:** Demographics summary of respondents.

Category	Subcategory	Frequency	Percentage (%)
Qualification	Bachelor’s	63	63%
	Master’s	21	21%
	PhD	16	16%
Experience (Years)	1–5	54	54%
	6–10	32	32%
	Above 10	14	14%
Background	Manager	72	72%
	Supervisor	18	18%
	Researcher	10	10%
Industry Type	Small Firm	32	32%
	Medium Firm	59	59%
	Large Firm	9	9%
Industry Sector	Automotive	34	34%
	Electronics and Electrical	25	25%
	Machinery and Equipment	18	18%
	Chemicals and Pharmaceuticals	14	14%
	Textiles	9	9%

**Table 6:** EFA results for the study.

Cluster	Challenges	Average	Loading	Communalities	Significance
Technological Challenges	Material Limitations	3.53	0.887	0.886	Yes
	Process Optimization	3.45	0.906	0.929	Yes
	Integration with AM	3.08	0.856	0.908	Yes
	Scalability Issues	3.9	0.885	0.918	Yes
Economic and Market Challenges	High Cost of Materials	3.4	0.907	0.919	Yes
	Market Readiness	3.48	0.901	0.924	Yes
	Cost-Effectiveness of AM	3.46	0.869	0.908	Yes
Regulatory and Standardization Challenges	Lack of Standardization	3.54	0.939	0.938	Yes
	Regulatory Compliance	3.38	0.901	0.929	Yes
	Supply Chain Constraints	3.19	0.874	0.926	Yes
Environmental and Sustainability Challenges	End-of-Life Management	3.17	0.899	0.875	Yes
	Energy Consumption	3.82	0.885	0.877	Yes
	Carbon Footprint	3.25	0.909	0.913	Yes
	LCA Assessment Gaps	3.93	0.905	0.896	Yes

**Table 7:** Challenge Clusters, Interventions and Stakeholders.

Challenge Cluster	Interventions	Primary Stakeholders
Technological	Develop tailored PBSM feedstocks Optimize AM process	R&D departments; AM equipment OEMs
Environmental and Sustainability	Deploy dedicated recycling streams Conduct cradle-to-grave LCAs	Recycling firms; LCA experts
Economic and Market	Offer government subsidies or tax incentives Create industry consortiums to aggregate demand	Policymakers; Industry associations; Investors
Regulatory and Standardization	Publish consensus test methods Harmonize material certification	Standards bodies (ISO, ASTM); Regulators

**Table 8:** Initial decision-making matrix.

Experts	E1	E2	E3	E4	E5	E6
Weights	0.17	0.17	0.17	0.17	0.17	0.17
Material Limitations	4	3	3	5	3	4
Process Optimization	4	3	5	3	2	3
Integration with AM	4	3	4	3	3	1
Scalability Issues	3	4	4	4	2	2
High Cost of Materials	3	3	3	5	3	1
Market Readiness	4	3	4	5	5	3
Cost-Effectiveness of AM	4	3	4	4	4	5
Lack of Standardization	5	4	5	3	3	5
Regulatory Compliance	4	4	4	3	2	5
Supply Chain Constraints	3	3	4	4	3	2
End-of-Life Management	5	4	5	4	3	5
Energy Consumption	3	3	3	4	4	2
Carbon Footprint	3	4	4	5	3	5
LCA Assessment Gaps	4	4	3	4	3	2
MIN	3	3	3	3	2	1
MAX	5	4	5	5	5	5
RANGE	2	1	1	2	2	4

**Table 9:** Normalized matrix.

Challenges	E1	E2	E3	E4	E5	E6
Material Limitations	0.515	0.389	0.075	0.875	0.188	0.750
Process Optimization	0.730	0.411	0.992	0.000	0.000	0.500
Integration with AM	0.746	0.571	0.543	0.200	0.238	0.000
Scalability Issues	0.091	0.981	0.219	0.393	0.002	0.250
High Cost of Materials	0.126	0.644	0.115	1.000	0.217	0.000
Market Readiness	0.408	0.114	0.723	0.793	1.000	0.500
Cost-Effectiveness of AM	0.524	0.000	0.464	0.606	0.779	1.000
Lack of Standardization	0.991	0.651	0.970	0.184	0.550	1.000
Regulatory Compliance	0.365	0.879	0.405	0.092	0.050	1.000
Supply Chain Constraints	0.063	0.158	0.246	0.645	0.464	0.250
End-of-Life Management	1.000	0.987	1.000	0.561	0.535	1.000
Energy Consumption	0.077	0.374	0.000	0.705	0.642	0.250
Carbon Footprint	0.000	0.935	0.505	0.825	0.446	1.000
LCA Assessment Gaps	0.742	1.000	0.054	0.438	0.475	0.250

**Table 10:** Weighted normalized matrix.

Challenges	E1	E2	E3	E4	E5	E6	Si
Material Limitations	0.086	0.065	0.012	0.146	0.031	0.125	0.340
Process Optimization	0.122	0.069	0.165	0.000	0.000	0.083	0.356
Integration with AM	0.124	0.095	0.091	0.033	0.040	0.000	0.383
Scalability Issues	0.015	0.163	0.036	0.066	0.000	0.042	0.281
High Cost of Materials	0.021	0.107	0.019	0.167	0.036	0.000	0.350
Market Readiness	0.068	0.019	0.120	0.132	0.167	0.083	0.506
Cost-Effectiveness of AM	0.087	0.000	0.077	0.101	0.130	0.167	0.395
Lack of Standardization	0.165	0.108	0.162	0.031	0.092	0.167	0.558
Regulatory Compliance	0.061	0.147	0.068	0.015	0.008	0.167	0.298
Supply Chain Constraints	0.011	0.026	0.041	0.107	0.077	0.042	0.263
End-of-Life Management	0.167	0.164	0.167	0.093	0.089	0.167	0.680
Energy Consumption	0.013	0.062	0.000	0.117	0.107	0.042	0.300
Carbon Footprint	0.000	0.156	0.084	0.137	0.074	0.167	0.452
LCA Assessment Gaps	0.124	0.167	0.009	0.073	0.079	0.042	0.452
					min		0.263
					max		0.680
					sum		5.614

**Table 11:** Power of weighted compatibility matrix.

Challenges	E1	E2	E3	E4	E5	E6	Pi
Material Limitations	0.895	0.854	0.649	0.978	0.757	0.953	4.134
Process Optimization	0.949	0.862	0.999	0.000	0.000	0.891	2.810
Integration with AM	0.952	0.911	0.903	0.765	0.787	0.000	4.318
Scalability Issues	0.670	0.997	0.776	0.856	0.359	0.794	3.658
High Cost of Materials	0.708	0.929	0.697	1.000	0.775	0.000	4.109
Market Readiness	0.861	0.697	0.947	0.962	1.000	0.891	4.467
Cost-Effectiveness of AM	0.898	0.000	0.880	0.920	0.959	1.000	3.657
Lack of Standardization	0.998	0.931	0.995	0.754	0.905	1.000	4.584
Regulatory Compliance	0.845	0.979	0.860	0.672	0.606	1.000	3.962
Supply Chain Constraints	0.631	0.735	0.791	0.930	0.880	0.794	3.967
End-of-Life Management	1.000	0.998	1.000	0.908	0.901	1.000	4.807
Energy Consumption	0.653	0.849	0.000	0.943	0.929	0.794	3.374
Carbon Footprint	0.000	0.989	0.892	0.968	0.874	1.000	3.724
LCA Assessment Gaps	0.952	1.000	0.615	0.871	0.883	0.794	4.321
					min		2.810
					max		4.807
					sum		55.892

**Table 12:** Final ranking of the challenges.

Challenges	*K*_a_	Rank	*K_b_*	Rank	Lambda (*λ*)	0.5	*K*	Final Rank
					*K_c_*	Rank		
Material Limitations	0.073	6	2.767	9	0.815	6	1.766	8
Process Optimization	0.051	14	2.354	13	0.577	14	1.406	14
Integration with AM	0.076	5	2.995	6	0.857	5	1.890	5
Scalability Issues	0.064	12	2.372	12	0.718	12	1.529	12
High Cost of Materials	0.072	7	2.796	8	0.813	7	1.775	7
Market Readiness	0.081	3	3.518	3	0.906	3	2.138	3
Cost-Effectiveness of AM	0.066	11	2.807	7	0.738	11	1.719	9
Lack of Standardization	0.084	2	3.754	2	0.937	2	2.257	2
Regulatory Compliance	0.069	8	2.546	10	0.776	8	1.646	10
Supply Chain Constraints	0.069	9	2.412	11	0.771	9	1.588	11
End-of-Life Management	0.089	1	4.302	1	1.000	1	2.524	1
Energy Consumption	0.060	13	2.342	14	0.669	13	1.478	13
Carbon Footprint	0.068	10	3.045	5	0.761	10	1.831	6
LCA Assessment Gaps	0.078	4	3.257	4	0.870	4	2.005	4

## Data Availability

The data supporting the findings of this study are available upon request from the corresponding author.

## References

[1] Mahmood A., Perveen F., Chen S., Akram T., Irfan A. (2024). Polymer Composites in 3D/4D Printing: Materials, Advances, and Prospects. Molecules.

[2] Qureshi K.M., Yadav A., Garg R.K., Sachdeva A., Abdulrahman A., Alghamd S.Y., Qureshi M.R.N.M. (2024). Are Polymer-Based Smart Materials Unlocking the Path to Sustainable Manufacturing for a Net-Zero Economy? Current Trends and Potential Applications. IEEE Access.

[3] Yadav A., Sachdeva A.K., Agrawal R., Garg R.K. (2022). Environmental Sustainability of Additive Manufacturing: A Case Study of Indian Manufacturing Industry. Proceedings of the ASME International Mechanical Engineering Congress and Exposition.

[4] Yadav A., Garg R.K., Sachdeva A., Qureshi M.R.N.M. (2025). Comparing Environmental Sustainability of Additive Manufacturing and Investment Casting: Life Cycle Assessment of Aluminium LM04 (Al-Si5-Cu3). Mater. Sci. Eng. A.

[5] Zhou L., Miller J., Vezza J., Mayster M., Raffay M., Justice Q., Al Tamimi Z., Hansotte G., Sunkara L.D., Bernat J. (2024). Additive Manufacturing: A Comprehensive Review. Sensors.

[6] Zhang X., Liang E. (2019). Metal Additive Manufacturing in Aircraft: Current Application, Opportunities and Challenges. Proceedings of the IOP Conference Series: Materials Science and Engineering.

[7] Yuan S., Shen F., Chua C.K., Zhou K. (2019). Polymeric Composites for Powder-Based Additive Manufacturing: Materials and Applications. Prog. Polym. Sci..

[8] Al Rashid A., Koç M. (2023). Additive Manufacturing for Sustainability and Circular Economy: Needs, Challenges, and Opportunities for 3D Printing of Recycled Polymeric Waste. Mater. Today Sustain..

[9] Wu Y., Lu Y., Zhao M., Bosiakov S., Li L. (2022). A Critical Review of Additive Manufacturing Techniques and Associated Biomaterials Used in Bone Tissue Engineering. Polymers.

[10] Al Noman A., Kumar B.K., Dickens T. (2023). Field Assisted Additive Manufacturing for Polymers and Metals: Materials and Methods. Virtual Phys. Prototyp..

[11] De France K.J., Xu F., Hoare T. (2018). Structured Macroporous Hydrogels: Progress, Challenges, and Opportunities. Adv. Healthc. Mater..

[12] Armenta S., Esteve-Turrillas F.A., Garrigues S., de la Guardia M. (2021). Smart Materials for Sample Preparation in Bioanalysis: A Green Overview. Sustain. Chem. Pharm..

[13] Abouchenari A., Tajbakhsh N., Shahbaz A., Alamdari-Mahd G. (2024). Advancements in Dental Implant Technology: The Impact of Smart Polymers Utilized through 3D Printing. J. Synth. Sinter..

[14] Chen J., Virrueta C., Zhang S., Mao C., Wang J. (2024). 4D Printing: The Spotlight for 3D Printed Smart Materials. Mater. Today.

[15] Enyan M., Bing Z., Amu-Darko J.N.O., Issaka E., Otoo S.L., Agyemang M.F. (2025). Advances in Smart Materials Soft Actuators on Mechanisms, Fabrication, Materials, and Multifaceted Applications: A Review. J. Thermoplast. Compos. Mater..

[16] Hajare D.M., Gajbhiye T.S. (2022). Additive Manufacturing (3D Printing): Recent Progress on Advancement of Materials and Challenges. Mater. Today Proc..

[17] Jin L., Zhai X., Zhang K., Jiang J. (2025). Unlocking the Potential of Low-Melting-Point Alloys Integrated Extrusion Additive Manufacturing: Insights into Mechanical Behavior, Energy Absorption, and Electrical Conductivity. Prog. Addit. Manuf..

[18] Goh G.D., Yap Y.L., Agarwala S., Yeong W.Y. (2019). Recent Progress in Additive Manufacturing of Fiber Reinforced Polymer Composite. Adv. Mater. Technol..

[19] Deisenroth D.C., Moradi R., Shooshtari A.H., Singer F., Bar-Cohen A., Ohadi M. (2018). Review of Heat Exchangers Enabled by Polymer and Polymer Composite Additive Manufacturing. Heat Transf. Eng..

[20] Arshad M.U. (2024). Exploring the Latest Advances in Materials Science: Development of New Materials with Unique Properties. Soc. Sci. Rev. Arch..

[21] Vicente C.M.S., Sardinha M., Reis L., Ribeiro A., Leite M. (2023). Large-Format Additive Manufacturing of Polymer Extrusion-Based Deposition Systems: Review and Applications. Prog. Addit. Manuf..

[22] Yang Y., Song X., Li X., Chen Z., Zhou C., Zhou Q., Chen Y. (2018). Recent Progress in Biomimetic Additive Manufacturing Technology: From Materials to Functional Structures. Adv. Mater..

[23] Radu I.-C., Vadureanu A.-M., Cozorici D.-E., Blanzeanu E., Zaharia C. (2025). Advancing Sustainability in Modern Polymer Processing: Strategies for Waste Resource Recovery and Circular Economy Integration. Polymers.

[24] Sarabia-Vallejos M.A., Rodríguez-Umanzor F.E., González-Henríquez C.M., Rodríguez-Hernández J. (2022). Innovation in Additive Manufacturing Using Polymers: A Survey on the Technological and Material Developments. Polymers.

[25] Salifu S., Ogunbiyi O., Olubambi P.A. (2022). Potentials and Challenges of Additive Manufacturing Techniques in the Fabrication of Polymer Composites. Int. J. Adv. Manuf. Technol..

[26] Rahman M., Islam K.S., Dip T.M., Chowdhury M.F.M., Debnath S.R., Hasan S.M.d.M., Sakib M.d.S., Saha T., Padhye R., Houshyar S. (2024). A Review on Nanomaterial-Based Additive Manufacturing: Dynamics in Properties, Prospects, and Challenges. Prog. Addit. Manuf..

[27] Leung Y.-S., Kwok T.-H., Li X., Yang Y., Wang C.C., Chen Y. (2019). Challenges and Status on Design and Computation for Emerging Additive Manufacturing Technologies. J. Comput. Inf. Sci. Eng..

[28] Bean R.H., Long T.E. (2024). Recent Trends in the Additive Manufacturing of Polyurethanes. Polym. Int..

[29] Harun-Ur-Rashid M., Jahan I., Islam M.J., Kumer A., Huda M.N., Imran A.B., Gouadria S., Alsalhi S.A. (2024). Global Advances and Smart Innovations in Supramolecular Polymers. J. Mol. Struct..

[30] Jyothika M., Gowda S.M.J., Kumar G.B.K., Rakshitha K.B., Srikruthi K.S., Goudanavar P., Naveen N.R. (2024). Innovative Polymer Modifications: Unlocking New Horizons in Drug Delivery Applications. Biomed. Mater. Devices.

[31] Olawumi M.A., Omigbodun F.T., Oladapo B.I. (2024). Integration of Sustainable and Net-Zero Concepts in Shape-Memory Polymer Composites to Enhance Environmental Performance. Biomimetics.

[32] Kantaros A., Ganetsos T. (2023). From Static to Dynamic: Smart Materials Pioneering Additive Manufacturing in Regenerative Medicine. Int. J. Mol. Sci..

[33] Zheng J., Qiu X., Zhu J., Jia R., Xu B., Xia Z., Wang W., Ling W., Tang B., Guan A. (2025). Improved Forming Efficiency Through the Adjustment of Inkjet-Bonded 3D Printing Parameters and Sand Mold Structure. Int. J. Met..

[34] Attri A., Yadav A., Garg R.K., Bhardwaj A., Pandey P.M., Misra A. (2024). Enhancing Supply Chain Sustainability Through Industry 4.0 and Additive Manufacturing Technologies: A Bibliometric-Based Review. Optimization of Production and Industrial Systems.

[35] Ligon S.C., Liska R., Stampfl J., Gurr M., Mülhaupt R. (2017). Polymers for 3D Printing and Customized Additive Manufacturing. Chem. Rev..

[36] Tan L.J., Zhu W., Zhou K. (2020). Recent Progress on Polymer Materials for Additive Manufacturing. Adv. Funct. Mater..

[37] Prudente I.N.R., dos Santos H.C., Fonseca J.L., de Almeida Y.A., Gimenez I.d.F., Barreto L.S. (2024). Graphene Family (GFMs), Carbon Nanotubes (CNTs) and Carbon Black (CB) on Smart Materials for Civil Construction: Self-Cleaning, Self-Sensing and Self-Heating. J. Build. Eng..

[38] Nhu T.T., Boone L., Guillard V., Chatellard L., Reis M., Matos M., Dewulf J. (2024). Environmental Sustainability Assessment of Biodegradable Bio-Based Poly(3-Hydroxybutyrate-*Co*-3-Hydroxyvalerate) from Agro-Residues: Production and End-of-Life Scenarios. J. Environ. Manag..

[39] Irfan J., Ali A., Hussain M.A., Haseeb M.T., Naeem-ul-Hassan M., Hussain S.Z. (2024). Citric Acid Cross-Linking of a Hydrogel from *Aloe Vera* (*Aloe Barbadensis* M.) Engenders a pH-Responsive, Superporous, and Smart Material for Drug Delivery. RSC Adv..

[40] Carvalho R., Lanceros-Mendez S., Martins P. (2024). Tailoring Polymer-Based Magnetoelectrics for Spintronics: Evaluating the Converse Effect. Appl. Mater. Today.

[41] Kadapure S.A., Deshannavar U.B. (2022). Bio-Smart Material in Self-Healing of Concrete. Mater. Today Proc..

[42] Maidin S., Wee K.J., Sharum M.A., Rajendran T.K., Ali L.M., Ismail S. (2023). A Review on 4D Additive Manufacturing-the Applications, Smart Materials & Effect of Various Stimuli on 4D Printed Objects. J. Teknol..

[43] Chakroborty S., Nath N., Mahal A., Barik A., Panda A.R., Fahaduddin, Bal T., Obaidullah A.J.;, Elawady A. (2024). Stimuli-Responsive Nanogels: A Smart Material for Biomedical Applications. J. Mol. Liq..

[44] Saleh Alghamdi S., John S., Roy Choudhury N., Dutta N.K. (2021). Additive Manufacturing of Polymer Materials: Progress, Promise and Challenges. Polymers.

[45] Patti A. (2024). Challenges to Improve Extrusion-Based Additive Manufacturing Process of Thermoplastics toward Sustainable Development. Macromol. Rapid Commun..

[46] Prasad G., Arunav H., Dwight S., Ghosh M.B., Jayadev A., Nair D.I. (2024). Advancing Sustainable Practices in Additive Manufacturing: A Comprehensive Review on Material Waste Recyclability. Sustainability.

[47] Franco Urquiza E.A. (2024). Advances in Additive Manufacturing of Polymer-Fused Deposition Modeling on Textiles: From 3D Printing to Innovative 4D Printing—A Review. Polymers.

[48] Nasr Azadani M., Zahedi A., Bowoto O.K., Oladapo B.I. (2022). A Review of Current Challenges and Prospects of Magnesium and Its Alloy for Bone Implant Applications. Prog. Biomater..

[49] Shelare S., Aglawe K., Giri S., Waghmare S. (2023). Additive Manufacturing of Polymer Composites: Applications, Challenges and Opportunities. Indian J. Eng. Mater. Sci. (IJEMS).

[50] Agrawal R., Priyadarshinee P., Kumar A., Luthra S., Garza-Reyes J.A., Kadyan S. (2023). Are Emerging Technologies Unlocking the Potential of Sustainable Practices in the Context of a Net-Zero Economy? An Analysis of Driving Forces. Environ. Sci. Pollut. Res..

[51] Field A. (2013). Discovering Statistics Using IBM SPSS Statistics.

[52] Reio T.G., Shuck B. (2015). Exploratory Factor Analysis: Implications for Theory, Research, and Practice. Adv. Dev. Hum. Resour..

[53] Jamwal A., Kumari S., Agrawal R., Sharma M., Gölgeci I. (2024). Unlocking Circular Economy Through Digital Transformation: The Role of Enabling Factors in SMEs. JGBC.

[54] Der O., Ordu M., Başar G. (2024). Multi-Objective Optimization of Cutting Parameters for Polyethylene Thermoplastic Material by Integrating Data Envelopment Analysis and SWARA-Based CoCoSo Approach. Osman. Korkut Ata Üniversitesi Fen Bilim. Enstitüsü Derg..

[55] Kumar V., Kalita K., Chatterjee P., Zavadskas E.K., Chakraborty S. (2022). A SWARA-CoCoSo-Based Approach for Spray Painting Robot Selection. Informatica.

